# Proteomic Analysis of Lysine Acetylation and Succinylation to Investigate the Pathogenicity of Virulent *Pseudomonas syringae* pv. *tomato* DC3000 and Avirulent Line *Pseudomonas syringae* pv. *tomato* DC3000 *avrRpm1* on *Arabidopsis thaliana*

**DOI:** 10.3390/genes15040499

**Published:** 2024-04-16

**Authors:** Yongqiang Ding, Yangxuan Liu, Kexin Yang, Yiran Zhao, Chun Wen, Yi Yang, Wei Zhang

**Affiliations:** 1 Key Laboratory of Bio-Resources and Eco-Environment of Ministry of Education, College of Life Sciences, Sichuan University, Chengdu 610065, China; dyq910420@163.com (Y.D.); y18284319800@163.com (K.Y.); zyr7129219@163.com (Y.Z.); wenchungo@163.com (C.W.); yangyi528@scu.edu.cn (Y.Y.); 2 School of Life Sciences, Jiangsu University, Zhenjiang 212013, China; 3 National Key Laboratory of Plant Molecular Genetics, CAS Center for Excellence in Molecular Plant Sciences, Institute of Plant Physiology and Ecology, Chinese Academy of Sciences, Shanghai 200032, China; yxliu01@cemps.ac.cn

**Keywords:** *Pst* DC3000, *Pst-avrRpm1*, lysine acetylation, lysine succinylation, proteomic analysis, pathogenicity

## Abstract

*Pseudomonas syringae* pv. *tomato* DC3000 (*Pst* DC3000) is able to infect many economically important crops and thus causes substantial losses in the global agricultural economy. *Pst* DC3000 can be divided into virulent lines and avirulent lines. For instance, the pathogen effector avrRPM1 of avirulent line *Pst-avrRpm1* (*Pst* DC3000 *avrRpm1*) can be recognized and detoxified by the plant. To further compare the pathogenicity mechanisms of virulent and avirulent *Pst* DC3000, a comprehensive analysis of the acetylome and succinylome in *Arabidopsis thaliana* was conducted following infection with virulent line *Pst* DC3000 and avirulent line *Pst-avrRpm1*. In this study, a total of 1625 acetylated proteins encompassing 3423 distinct acetylation sites were successfully identified. Additionally, 229 succinylated proteins with 527 unique succinylation sites were detected. A comparison of these modification profiles between plants infected with *Pst* DC3000 and *Pst-avrRpm1* revealed significant differences. Specifically, modification sites demonstrated inconsistencies, with a variance of up to 10% compared to the control group. Moreover, lysine acetylation (Kac) and lysine succinylation (Ksu) displayed distinct preferences in their modification patterns. Lysine acetylation is observed to exhibit a tendency towards up-regulation in *Arabidopsis* infected with *Pst-avrRpm1*. Conversely, the disparity in the number of Ksu up-regulated and down-regulated sites was not as pronounced. Motif enrichment analysis disclosed that acetylation modification sequences are relatively conserved, and regions rich in polar acidic/basic and non-polar hydrophobic amino acids are hotspots for acetylation modifications. Functional enrichment analysis indicated that the differentially modified proteins are primarily enriched in the photosynthesis pathway, particularly in relation to light-capturing proteins. In conclusion, this study provides an insightful profile of the lysine acetylome and succinylome in *A. thaliana* infected with virulent and avirulent lines of *Pst* DC3000. Our findings revealed the potential impact of these post-translational modifications (PTMs) on the physiological functions of the host plant during pathogen infection. This study offers valuable insights into the complex interactions between plant pathogens and their hosts, laying the groundwork for future research on disease resistance and pathogenesis mechanisms.

## 1. Introduction

Plants are frequently subjected to numerous pathogenic bacteria, which adversely affect their growth and productivity. These plant diseases result in significant crop losses, posing a persistent challenge to global food security [1]. Bacterial plant pathogens, such as *Pseudomonas*, *Ralstonia*, *Xanthomonas*, and *Erwinia*, have been known to cause a variety of diseases in important crop plants [2]. Over extended periods of coexistence, both hosts and pathogens have evolved intricate strategies to counter each other’s attacks [3]. The virulent strain *Pst* DC3000, serving as a model strain for elucidating plant–pathogen interactions, can induce bacterial speck disease in its host plants, compromising their growth, development, and reproductive capabilities [4,5]. To date, over 50 bacterial effector proteins have been identified from the *Pst* DC3000 strain [6]. The intricate interplay between *P. syringae* and plants holds immense significance for further exploring the specific molecular mechanisms underlying plant responses to pathogenic bacteria and for developing effective control measures.

*Pst* DC3000 is a pivotal model for elucidating the functional mechanisms of diverse effectors. This strain secretes numerous effectors through the type III secretion system, including AvrPto and AvrPtoB, which play crucial roles in plant–pathogen interactions [6]. In resistant plant varieties, AvrPto and AvrPtoB are specifically recognized by the serine–threonine kinase Pto. This recognition triggers the activation of the NB-LRR protein Prf, leading to localized cell death and conferring resistance against the pathogen [7,8]. Additionally, two type III effectors, AvrRpm1 and AvrB, have been shown to interact with the RPM1-interacting protein 4 (RIN4), resulting in its phosphorylation [6]. AvrRpm1, as an ADP-ribosyltransferase, modifies the resistance protein RIN4 in *Arabidopsis*, ultimately activating the *Arabidopsis* protein RPM1. Similarly, AvrB is recognized in *Arabidopsis*, and the activation of RPM1 is closely associated with the phosphorylation of a conserved threonine residue (T166) within RIN4. Previous studies have demonstrated that the ADP-ribosylation of RIN4 by AvrRpm1 triggers the phosphorylation of T166, subsequently eliciting an RPM1-mediated defense response in the host plant [9]. Mutant strains exhibiting dysfunctional effectors are frequently employed in comparative studies involving pathogenic strains, including DC3000, to explore the functionalities of these effectors. For instance, the pathogen effector avrRPM1 of the avirulent line *Pst-avrRpm1* (*Pst* DC3000 *avrRpm1*) is frequently used in such comparisons. Understanding these intricate interactions between effectors and host proteins carries significant implications for developing novel strategies to combat plant diseases.

Emerging evidence emphasizes the critical role of protein PTMs in mediating plant–pathogen interactions [10,11]. Among the 20 canonical amino acid residues, lysine (K) residues are frequently subjected to various PTMs, collectively referred to as K-PTMs, which are among the most commonly observed modifications [12]. Notably, lysine residues are susceptible to a range of sequential or cascading covalent modifications, including acetylation, phosphorylation, crotonylation, butyrylation, propionylation, glutarylation, methylation, and succinylation. These diverse PTMs are hypothesized to play pivotal roles in regulating gene expression in microorganisms [13,14].

Lysine acetylation, a dynamic and reversible PTM tightly regulated by the balanced activities of acetyltransferases and deacetylases, is of particular interest [15]. This modification, commonly observed on histones and widespread among non-histone proteins as well [10,11], influences protein functions through multiple mechanisms, including modulating protein stability, subcellular localization, protein–protein interactions, and crosstalk with other PTMs [15]. Given its widespread occurrence and diverse functional implications, lysine acetylation represents a compelling target for future investigations aimed at elucidating the complex mechanisms underlying plant–pathogen interactions.

The amino group of lysine serves as a diverse substrate for various modifications, including acetylation, acylations, methylation, ubiquitylation, and ubiquitin-like modifiers. This versatility gives rise to competitive PTM crosstalk, where different PTMs compete for the same lysine residue [16]. Proteomic investigations have revealed that a significant proportion of acetylated lysines are also targets of other PTMs, such as ubiquitylation and succinylation [17,18]. Previous studies have elucidated that the interplay between lysine acetylation and succinylation modulates bacterial quorum sensing [19].

Succinylation, a recently discovered reversible PTM, entails the transfer of a succinyl group from succinyl-CoA to the ε-amino group of a specific lysine residue, leading to the formation of succinyl-lysine [20,21]. Succinylation introduces a larger structural moiety compared to acetylation or methylation, potentially leading to more significant alterations in protein structure and function [20,21]. However, the mechanisms underlying the interaction between succinylation and other metabolic pathways remain elusive. Further exploration is required to deepen our understanding of the regulatory mechanisms governing succinylation, and there is a pressing need for the functional validation of succinylation-related genes. Previous studies have reported the presence of acetylation [22] and succinylation [23] modifications in *Candida albicans*. Acetylation has been shown to regulate the stability of effectors in fungal plant pathogens, thereby affecting virulence [10]. Nevertheless, the impact of lysine acetylation and succinylation on the pathogenicity of *Pst* DC3000 remains largely unexplored.

In this study, high-resolution liquid chromatography–mass spectrometry (LC-MS/MS) was employed to investigate the lysine acetylation and succinylation profiles of *A. thaliana* infected with virulent *Pst* DC3000 and avirulent *Pst-avrRpm1*. The results revealed a widespread occurrence of these modifications, with a total of 3423 acetylation sites identified within 1625 proteins and 527 succinylation sites within 229 proteins. To gain further insights, the functions of the substrate proteins subject to acetylation and succinylation were categorized, and the local amino acid sequences surrounding the acetylated and succinylated lysine residues were analyzed. These findings provide valuable insights into the role of lysine acetylation and succinylation and their potential impact on the pathogenicity of *Pst* DC3000.

## 2. Materials and Methods

### 2.1. Plant Materials and Growth Conditions

The Arabidopsis thaliana ecotype Columbia (Col-0) served as the wild-type control in this study. Seeds of Col-0 were obtained from the Arabidopsis Biological Resource Center (ABRC) at Ohio State University, Columbus, OH, USA. Plants were cultivated in a greenhouse maintained at optimal conditions for their growth and development, with a temperature of 22/20 °C (day/night) and a photoperiod of 14/10 h (light/dark).

### 2.2. Inoculation of A. thaliana with Pst DC3000

The pathogenic bacterium *Pst* DC3000 and an avirulent pathogen *Pst-avrRpm1* were used in this study. They were provided by WenMing Wang from Sichuan Agricultural University [24]. *Pst* DC3000 and *Pst-avrRpm1* were propagated in liquid King’s B medium supplemented with rifampicin to ensure the purity of the culture. When the bacterial growth reached the late log phase of growth (OD_600_ = 0.6–1.0), bacteria were diluted to the required concentration in 10 mM MgCl_2_. Bacteria were diluted to OD_600_ = 0.2 (~1 × 10^8^ cfu mL^−1^) and sprayed onto plants until dripping according to the literature [24]. Following a 24 h incubation in the dark in a growth chamber, the plants were returned to their regular growth conditions. Disease symptoms were monitored daily, and samples were collected when phenotypic differences became apparent.

### 2.3. LC-MS/MS Analysis of Lysine Acetylation and Succinylation

To investigate lysine acetylation and succinylation in *Arabidopsis* infected with *Pst* DC3000 and *Pst-avrRpm1*, the tryptic peptides were dissolved in solvent A (consisting of 0.1% formic acid in 2% acetonitrile and 98% water). The peptides were then separated using the EASY-nLC 1000 system (Thermo, Saint Louis, MO, USA) at a flow rate of 700 nL/min. A gradient elution with solvent B (0.1% formic acid in 90% acetonitrile) was employed, increasing from 8% to 23% over 36 min, followed by a rise to 35% over 18 min, and maintained at 80% for 3 min. Chromatographic separation was performed on a custom-made analytical column, featuring an integrated spray tip (150 μm internal diameter, 20 cm length), packed with 1.9 μm/120 Å ReproSil-Pur C18 resins (Dr. Maisch GmbH, Ammerbuch, Germany).

### 2.4. Motif Identification and Residues Heat Map

Pre-aligned modification site windows of 21 residues (centered at K ± 10 residues) were input into MOMO to obtain motifs. MEME was subsequently utilized to identify overrepresented motifs with a high-frequency lysine. For MEME, the peptide windows were converted to FASTA format and input as a single peptide enriched with target motifs. MEME was also employed to generate a matrix of residue counts (position frequency matrix) for sequence windows centered on acetylated lysine, which was then used to perform Fisher’s exact test. The filtered *p*-value matrix was standardized using the function x = −log_10_ (*p*-value). Subsequently, the x values were z-transformed for each substrate category, ensuring a normalized representation across all categories. The z scores were clustered by one-way hierarchical clustering (Euclidean distance, average linkage clustering) in Genesis. Cluster membership was visualized by a heat map using the “heatmap.2” function from the “gplots” R-package (version 3.1.3.1).

### 2.5. Functional Enrichment Analysis

Functional enrichment analysis was conducted using the DAVID Bioinformatics Resources 6.7 platform [25]. This analysis aimed to identify enriched Gene Ontology (GO) terms, Kyoto Encyclopedia of Genes and Genomes (KEGG) IDs, and domains within our dataset. The significance of enrichment was evaluated using a two-tailed Fisher’s exact test, comparing the protein-containing international protein index (IPI) entries against all IPI proteins. To account for multiple hypothesis testing, standard false discovery rate control methods were applied. Terms with a corrected *p*-value < 0.05 were considered statistically significant.

## 3. Results

### 3.1. Systematic Identification of Lysine Acetylome and Succinylation in A. thaliana

**Figure 1:**
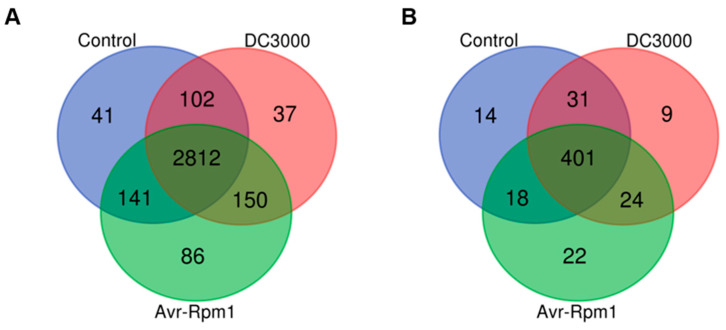
Venn diagrams of the identified acetylated (**A**) and succinylated (**B**) sites. The blue color represents the control, while the red and green color represent *Arabidopsis* leaf samples infected with *Pst* DC3000 and *Pst-AvrRpm1*, respectively. The numerical values correspond to the quantity of lysine modification sites in each category.

Insights into the lysine acetylome and succinylation in *A. thaliana* were sought through a proteomic analysis of plants infected with *Pst* DC3000 and *Pst-avrRpm1* using LC-MS. The site-specific mass error data were further analyzed in MATLAB. Scatter plots were generated to visualize the distribution of mass errors for acetylation and succinylation sites (Figure S1). Notably, the mass error distribution for lysine acetylation peptides clustered tightly within 6 ppm, whereas the mass error distribution for lysine succinylation peptides was even narrower, falling below 4 ppm. These results underscore the high quality and precision of our MS data, suitable for subsequent bioinformatics analysis. A two-dimensional distribution plot was constructed to assess the lengths of the cleaved peptide fragments. The plot revealed that the majority of peptides had lengths ranging from 7 to 20 amino acids (Figure S2). Specifically, 78.48% of acetylated peptides and a remarkable 88.98% of succinylated peptides fell within this range, reflecting the characteristic patterns of trypsin digestion. Employing this comprehensive proteomic approach, 3423 lysine acetylome (Kac) sites distributed across 1625 proteins (Figure 1A) and 527 lysine succinylation (Ksu) sites on 229 proteins (Figure 1B) were identified. To further explore the distribution of acetylation and succinylation sites, the number of modification sites per protein was analyzed. As shown in Figure S3, acetylation modifications were predominantly found at a single lysine residue in 58% of proteins, while 18% of proteins exhibited modifications at two lysine residues. The remaining proteins displayed modifications at three or more lysine residues, with a maximum of 22 Kac modification sites observed. In contrast, succinylation modifications exhibited a similar distribution pattern, with 50% of proteins modified at a single lysine residue, 19% modified at two lysine residues, and 15% modified at three or more lysine residues.

**Figure 2:**
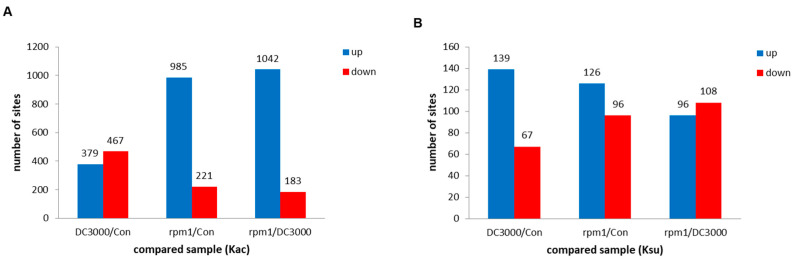
Column diagram depicting the distribution of Kac (**A**) and Ksu (**B**) sites across distinct comparison groups. Con represents the control, while DC3000 and rpm1 represent *Arabidopsis* leaf samples infected with *Pst* DC3000 and *Pst-AvrRpm1*, respectively. Kac: lysine acetylation; Ksu: lysine succinylation. Blue indicates the up while red indicates down.

To identify the differential modification sites, a threshold for relative abundance ratios was established. The relative abundance ratio of modification sites was calculated by the intensity ratio of peptides containing acetylation or succinylation separately in each sample compared to the control. Ratios exceeding 1.3-fold were deemed indicative of significant up-regulation, while ratios less than 1/1.3 were considered to represent a significant down-regulation. Upon comparing the DC3000/Con samples, no significant disparity was observed in the counts of up-regulated Kac sites (379) versus down-regulated sites (467) (Figure 2A). Interestingly, a distinct trend was observed in the rmp1/Con and rmp1/DC3000 samples. Here, the count of up-regulated Kac sites was markedly higher than that of the down-regulated sites, with approximately 4.5-fold and 5-fold differences, respectively (Figure 2A).

However, in the case of Ksu sites, a notable finding emerged that the number of up-regulated sites was twice that of the down-regulated ones between DC3000/Con (Figure 2B). Conversely, the disparity in the number of up-regulated and down-regulated Ksu sites in the rmp1/Con and rmp1/DC3000 samples was not as pronounced (Figure 2B). These findings suggest that upon infection with *Pst-avrRpm1*, *Arabidopsis* plants tend towards an up-regulation of lysine acetylation. A similar, albeit less pronounced, pattern was also observed for succinylation.

### 3.2. Motif Analysis of Lysine Acetylation and Succinylation Sites

To gain deeper insights into the characteristics of lysine acetylation and succinylation sites in *Arabidopsis*, the Motif-x program was employed to scrutinize the sequence motifs present in the identified peptides. This comprehensive analysis revealed 21 conserved acetylation motifs, encompassing 2681 acetylation modification sites, which accounted for a substantial 78.32% of the total Kac sites. These 21 motifs are summarized and described as follows (Kac represents the modified site, and the subscript indicates the relative position of the amino acid):

A_−1_\F_−1_\G_−1_\L_−1_\P_−1_\V_−1_\Y_−1_K_ac_N_+1_
K_ac_F_+1_\N_+1_\S_+1_\T_+1_\Y_+1_\H_+1_\R_+1_\R_+2_
K_ac_K_+1\+5\+7_
A_-2_E_-2_K_ac_P_+2_
K_ac_K_+1_A_+2_
Y_−1_K_ac_.

**Figure 3:**
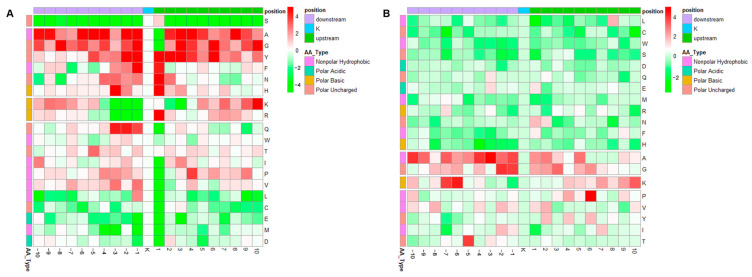
Motif analysis of the detected lysine acetylation (Kac) and lysine succinylation (Ksu) sites. Heat map analysis of the amino acid compositions around the acetylated (**A**) and succinylated (**B**) sites. Red indicates an amino acid that is significantly enriched, while green indicates an amino acid that is significantly reduced.

Furthermore, to facilitate a more intuitive understanding of the amino acid distributions around the acetylation sites, a heatmap was generated based on motif logo clustering analysis. This heatmap provides a visual representation of the frequency of amino acids spanning 10 positions upstream and downstream of the acetylation sites (Figure 3A). Notably, non-polar amino acids such as alanine (A), leucine (L), proline (P), and valine (V) exhibited significant enrichment in the −1 position, with the exception of glycine (G). Intriguingly, asparagine (N) consistently showed enrichment in the +1 position in this context. Additionally, polar amino acids, including asparagine (N), serine (S), threonine (T), and histidine (H), along with basic amino acids, were found to be significantly enriched in the +1 position. Lysine (K) demonstrated enrichment in the +1, +5, and +7 positions surrounding the acetylated lysine. Both phenylalanine (F) and tyrosine (Y) exhibited significant enrichment upstream and downstream of the acetylation sites. Additionally, the heatmap analysis unveils a noteworthy enrichment of alanine (A), glycine (G), and lysine (K) within Ksu (Figure 3B). Proline and threonine demonstrate elevated frequencies in the +6 and −5 positions, respectively.

### 3.3. Functional Annotation of Lysine-Acetylated and Lysine-Succinylated Proteins

**Figure 4:**
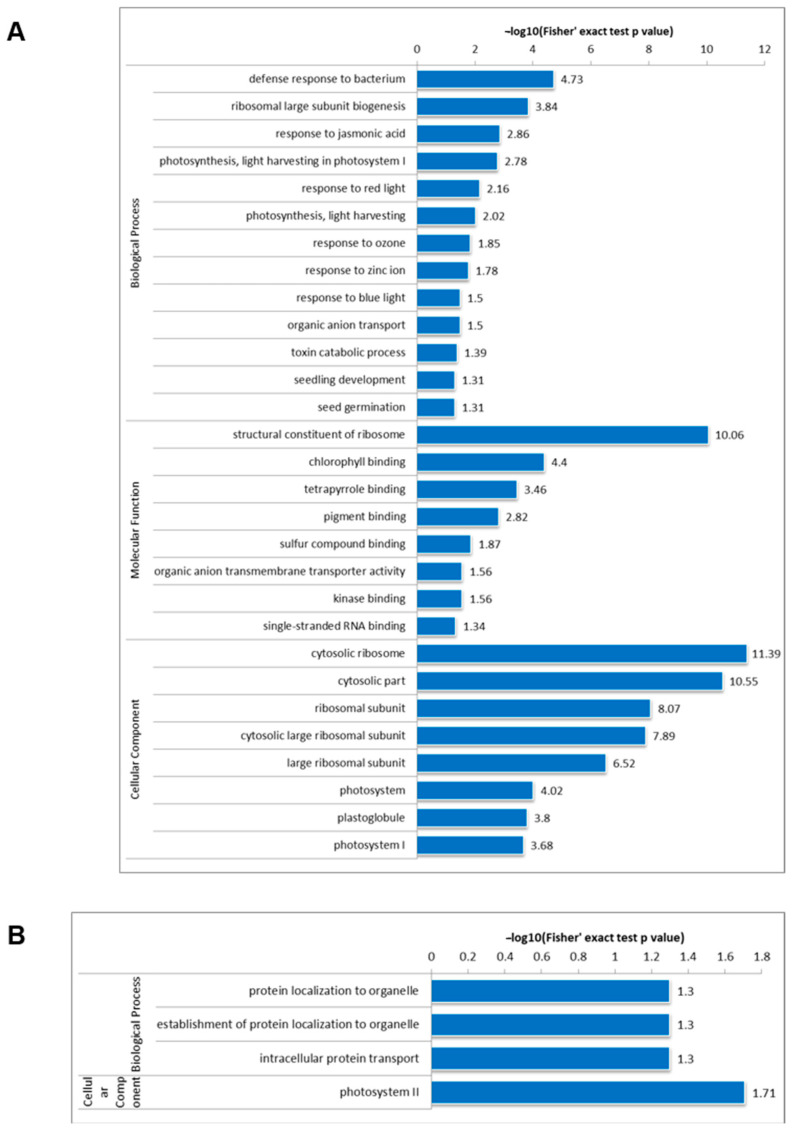
GO-based enrichment analysis of identified proteins. Enrichment analyses of the identified lysine-acetylated (**A**) and lysine-succinylated (**B**) proteins in the GO annotation and pathway categories. GO: Gene Ontology; BP: Biological Process; MF: Molecular Function; CC: Cellular Component.

To gain deeper insights into the functional roles of the identified acetylated sites, GO analyses were conducted in DC3000/rpm1. The results reveal that Kac proteins exhibit significant enrichment in bacterial defense responses and ribosomal large subunit biogenesis within the Biological Process (BP) category (Figure 4A). Within the Molecular Function (MF) category, ribosome structure constitution, chlorophyll binding, and tetrapyrrole binding are notably enriched. Regarding the Cellular Component (CC) category, cytosol, ribosome, and photosystem emerge as significantly enriched terms.

Turning attention to succinylation-differentially modified proteins, significant enrichment is observed in intracellular protein transport, protein localization to organelles, and the establishment of organelles within the BP category (Figure 4B). Notably, the photosystem II term stands out as significantly enriched within the CC category. Surprisingly, no specific categories are enriched within the MF category for these proteins (Figure 4B).

When focusing exclusively on proteins down-regulated in DC3000 compared to rpm1, a more pronounced enrichment of Ksu modifications is observed. This suggests that these down-regulated proteins harbor more specific and unique functional enrichments. Mirroring the enrichment patterns seen in all Kac-differentially modified proteins, the BP category is enriched in amide or peptide biosynthesis and biogenesis of the large ribosomal subunit. Within the MF category, ribosomal structural components, mRNA binding, and rRNA binding are enriched. Additionally, ribosomes and their S subunits are enriched in the CC category, all pointing to roles related to protein biosynthesis (Figure S4A).

Likewise, the enrichment analysis of distinct succinylated proteins hints at their involvement in photosynthesis. Specifically, in Figure S4B, the MF category is enriched in oxidoreductase activity, antioxidant activity, and peroxidase activity, while the CC category is enriched in mitochondria. These findings suggest potential roles for these proteins in redox reactions and respiration. Furthermore, the BP category is enriched in cell maturation, plant organ morphogenesis, and plant epidermal cell differentiation, implicating these proteins in plant growth processes.

### 3.4. KEGG Pathway Enrichment Analysis

**Figure 5:**
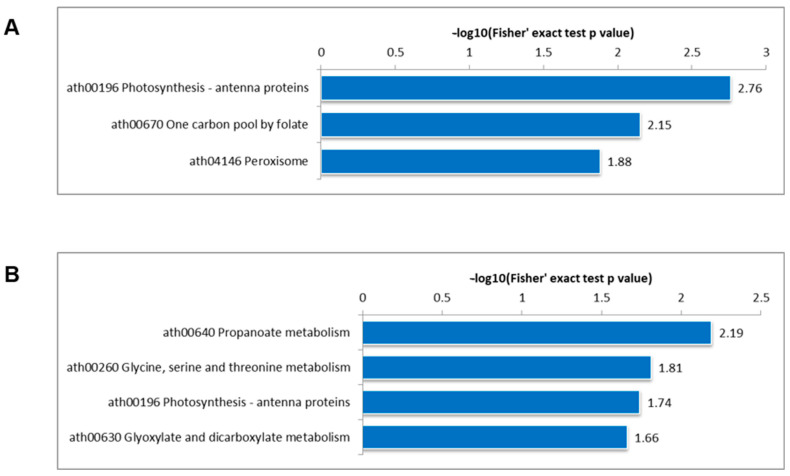
Enrichment analysis of KEGG pathways associated with down-regulated differentially modified Kac (**A**) and Ksu (**B**) proteins in *A. thaliana*. The significance of enrichment was evaluated using a two-tailed Fisher’s exact test. Terms with a corrected *p*-value < 0.05 were considered statistically significant.

To deepen the understanding of the metabolic regulation mediated by protein acetylation and succinylation, KEGG pathway enrichment analysis was conducted in DC3000/rpm1. The findings reveal that Kac-differentially modified proteins are enriched in the folate one-carbon pool and peroxisome pathways (Figure 5A). Meanwhile, proteins modified by Ksu exhibit enrichment in propanoate metabolism, glycine, serine, and threonine metabolism, as well as glyoxylate and dicarboxylate metabolism (Figure 5B). Notably, both PTMs show enrichment in photosynthesis-related antenna proteins. These results suggest that photosynthesis and carbohydrate metabolism are particularly susceptible to regulation by acetylation and succinylation, underscoring their critical roles in plant cellular metabolism.

### 3.5. Protein Domain Analysis

**Figure 6:**
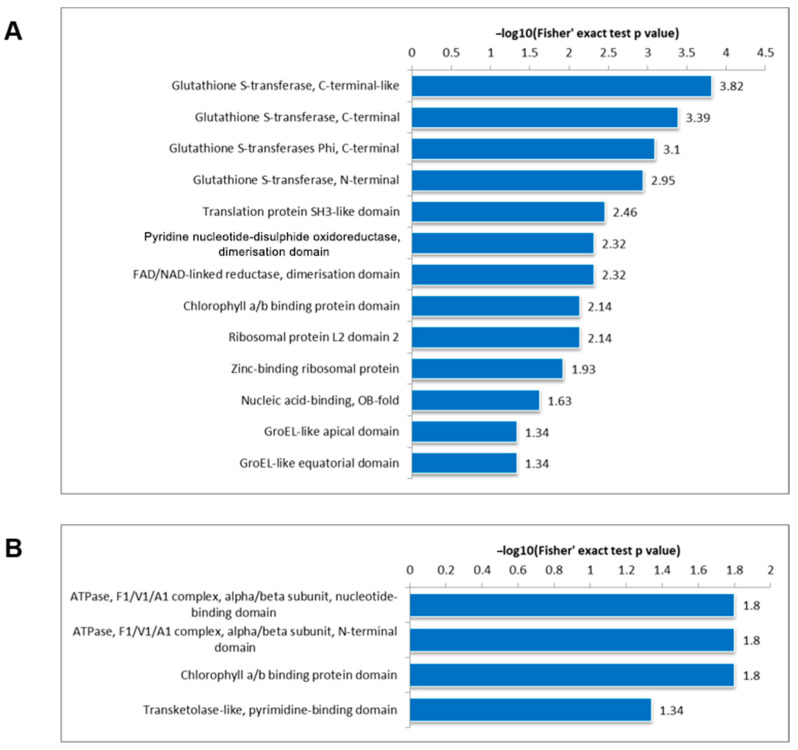
Protein domain enrichment analysis of acetylated (**A**) and succinylated (**B**) proteins. The significance of enrichment was evaluated using a two-tailed Fisher’s exact test. Terms with a corrected *p*-value < 0.05 were considered statistically significant.

The protein domain analysis conducted in DC3000/rpm1 provided intriguing insights into the modifications of Kac and Ksu. The findings indicate that both Kac and Ksu modifications are enriched in the chlorophyll a/b binding protein domain, a key component of photosynthesis. Kac-modified proteins also exhibited enrichment in glutathione S-transferase domains, translation protein SH3-like domains, ribosomal protein L2 domain 2, and zinc-binding ribosomal proteins (Figure 6A). Conversely, Ksu-modified proteins were enriched in ketol-like, pyrimidine binding domains; ATPase N-terminal domains; and nucleotide binding domains (Figure 6B).

**Figure 7:**
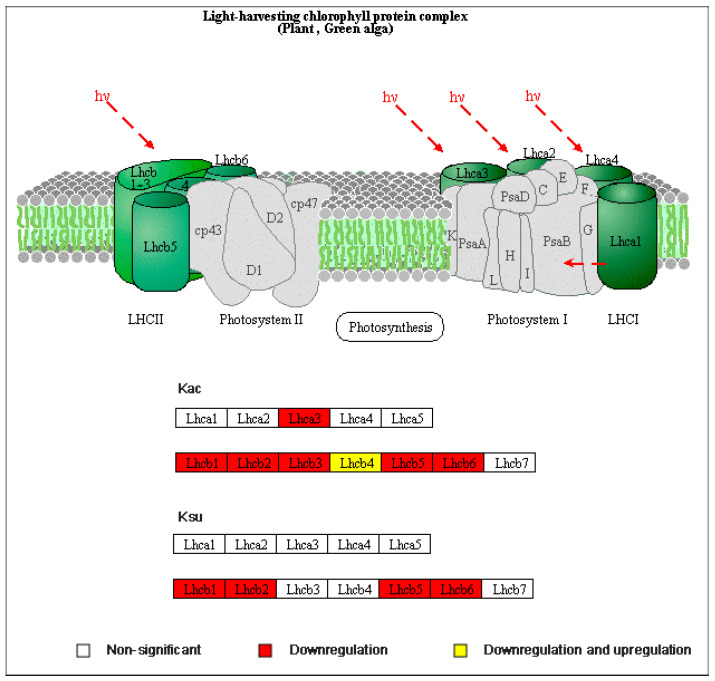
Detailed information on the photosynthesis-antenna protein pathway involving differentially modified Kac and Ksu proteins. The detected subunits of the light-harvesting chlorophyll protein complex are labeled with fold. White indicates non-significant change, red indicates down-regulation, while the yellow indicates both up-regulation and down-regulation.

Furthermore, a detailed examination of photosynthesis-antenna proteins commonly enriched in Kac and Ksu (Figure 7) revealed that acetylated and succinylated proteins are present in the light-harvesting chlorophyll protein complex (LHC), with a majority of them being down-regulated in the DC3000 VS rpm1 comparison. Notably, lhcb1, lhcb2, lhcb5, and lhcb6 underwent both Kac and Ksu modifications, which demonstrated synergistic effects of these two PTMs in photosynthesis. These findings suggest that Kac and Ksu play crucial roles in regulating photosynthesis and carbohydrate metabolism in plants, providing valuable insights into the complex metabolic networks that underlie plant growth and development.

## 4. Discussion

Biological stressors, particularly pathogenic bacterial infections, significantly impact the normal growth and developmental trajectories of plants, ultimately compromising crop yield and quality [26,27]. In the perpetual arms race between plants and pathogens, plants have evolved intricate defense mechanisms [28,29]. Notably, plants maintain a delicate balance between resisting pathogenic bacteria and supporting their own growth and development through a range of regulatory processes, including various protein PTMs. Lysine acetylation and succinylation emerge as prevalent PTMs in plants, playing pivotal roles in these regulatory networks [30,31].

In this study, a comprehensive analysis was undertaken to identify Kac and Ksu sites in *Arabidopsis* seedlings infected with Pst DC3000. A total of 3423 Kac sites on 1625 proteins (Figure 1A) and 527 Ksu sites on 229 proteins (Figure 1B) were identified. A comparative analysis between AIPD (*Arabidopsis* infected with *Pst* DC3000) and AIPA (*Arabidopsis* infected with *Pst-AvrRpm1*) samples revealed that 1225 Kac sites were unique, accounting for 41% of the total Kac sites. Similarly, a substantial proportion (48%) of Ksu sites were unique to these samples. While both AIPD and AIPA exhibited a subset of uniquely modified sites, these comprised less than 10% of the total sites identified. The majority of sites were consistently modified across all three sample groups. However, the abundance values of these co-modified sites varied, indicating a potential role for lysine acetylation and succinylation in mediating *Arabidopsis* resistance to *Pst* DC3000. Previous studies have reported on the Lys-acetylproteomes of plants in response to various biotic stresses, including pests, fungi, phytoplasma, and viruses [32,33,34,35]. Nonetheless, the concurrent regulation of plant disease resistance by lysine acetylation and succinylation remains underexplored. Our findings provide novel insights into the intricate regulatory networks underlying plant defense mechanisms and highlight the need for further investigation into the synergistic effects of these PTMs in mediating plant responses to biotic stress.

In the samples infected with *Pst-avrRpm1*/DC3000, an interesting pattern emerged: the number of Kac sites exhibiting up-regulation was more than fivefold greater than that down-regulated (Figure 2A). Meanwhile, the distribution of up- and down-regulated Ksu sites was relatively balanced (Figure 2B). Upon infection with *Pst-avrRpm1*, *Arabidopsis* exhibits a tendency towards increased lysine acetylation, accompanied by a comparable trend in succinylation, suggesting a coordinated modulation of these post-translational modifications in response to the pathogen. The marked contrast in acetylation patterns between AIPD and AIPA indicates that the two PTMs—acetylation and succinylation—may be differentially regulated in Arabidopsis during infection by distinct *Pst* strains.

Further analysis revealed 21 conserved acetylation motifs, encompassing 2681 Kac sites, which constitute 78.32% of the total identified Kac sites. Both the sequence logos and heatmaps clearly showed a preponderance of non-polar amino acids, polar uncharged amino acids, and basic amino acids (excluding the acidic amino acids Asp and Glu) in the vicinity of acetylation sites. This suggests that regions rich in polar acidic/basic and non-polar hydrophobic amino acids are hotspots for acetylation modifications. In a broader context, the acetylation of lysine residues serves to mask their positive charges, disrupting ionic and hydrogen bonding while increasing the hydrophobicity of proteins. This, in turn, can profoundly impact protein structure, function, and interactions with other cellular components, including DNA and proteins [36,37,38,39].

Intriguingly, despite concerted efforts, distinct succinylation modification motifs could not be identified. Nonetheless, analysis of the amino acid distribution around succinylated lysine residues revealed a similar pattern to that observed for acetylation: an enrichment of non-polar, polar basic, and polar uncharged amino acids, with acidic amino acids being underrepresented. Notably, the overall level of succinylation appeared to be lower than that of acetylation. Among the proteins that were found to be modified by both acetylation and succinylation, a significant overlap in modified sites within polar/basic and non-polar hydrophobic regions was detected [17,40,41]. These findings hint at a close relationship between acetylation and succinylation in plants, suggesting a possible synergistic role in mediating Arabidopsis’s response to biotic stress.

The enrichment patterns of down-regulated proteins mirror those observed for all differentially modified Kac proteins, pointing to their involvement in photosynthesis and bacterial defense mechanisms. Specifically, acetylated proteins exhibiting chlorophyll binding, pigment binding, and antioxidant activities predominantly populate the MF category. These proteins are primarily implicated in photosynthetic processes, particularly photorespiration. Correspondingly, photosynthesis-related terms such as light harvesting, response to red light, and response to blue light are enriched in the BP category, while photosystem components, notably photosystem I, are significantly enriched in the CC category. Proteins enriched in BP are predominantly linked to protein biosynthesis. Analogously, the enrichment of distinct succinylated proteins underscores their association with photosynthesis. Furthermore, the enrichment of oxidoreductase activity, antioxidant activity, peroxidase activity in MF, and mitochondria in CC hints at the potential roles of these proteins in redox reactions and respiration. Additionally, BP enrichment in cellular maturation, plant organ morphogenesis, and plant epidermal cell differentiation implicates these proteins in plant growth processes. These findings align with previous studies in Escherichia coli, suggesting that Ksu exhibits greater dynamism than Kac in response to alterations in growth conditions or genetic mutations. Previous reports have established the pivotal role of Kac and Ksu in regulating photosynthesis [30,31]. In conclusion, the results of GO enrichment analysis suggest that lysine acetylation and succinylation play crucial roles in Arabidopsis’s response to bacterial infection.

The enriched protein domain analysis revealed an intriguing finding: both lysine acetylation and succinylation modifications are enriched in the chlorophyll a/b binding protein domain. Chlorophyll a/b binding protein, particularly prevalent in higher plants, is a constituent of the light-harvesting chlorophyll a/b binding protein (LHCB). This LHCB protein plays a pivotal role as the apolipoprotein component of the photosystem II (PSII) light-harvesting complex, often in conjunction with chlorophyll and xanthophyll, functioning as a crucial antenna complex. Remarkably, LHCB may very well be one of the most abundant membrane proteins in nature, significantly bolstering photosynthetic efficiency.

Furthermore, our observations suggest that differences in Kac proteins are closely associated with biotic stress responses. This is evident from the plant’s response to bacteria and the enrichment of jasmonic acid in the BP category. Jasmonic acid, a key plant hormone, is well known for its crucial roles in mediating plant responses to various abiotic and biotic stresses, as well as regulating plant growth and development [42,43]. 

Partially infected plants develop systemic acquired resistance (SAR) and show heightened resistance during subsequent infections [44]. GLUTATHIONE-S-TRANSFERASE THETA 2 (GSTT2) GSTT2 plays an important role in SAR. GSTT2 expression increases in pathogen-inoculated as well as pathogen-free distal tissues. The loss-of-function mutant of GSTT2 activates normal local resistance, and *gstt2* mutant plants accumulate an enhanced level of methylated and acetylated histones in the promoters of WRKY6 and WRKY29 genes [44]. However, in our study, the protein domain analysis revealed that Kac-modified proteins also exhibited enrichment in glutathione S-transferase domains (Figure 6A). This means that the acetylation on Glutathione-S-transferase might inactivate its activity, since GSTT2 obviously plays a negative role in plant defense. Furthermore, GSST2 might regulate plant defense via regulating the acetylation of other defense-regulating genes such as *WRKR6* and *WKRY29*.

Notably, while lysine acetylation and succinylation are widely implicated in numerous central metabolic processes, including glycolysis, gluconeogenesis, and the citric acid cycle in diverse organisms such as plants, bacteria, and mammals [30,45], these core metabolic pathways were not enriched in the differentially modified proteins identified in our study. This finding suggests that the pathogenic differences observed between *Arabidopsis* infected with *Pst* DC3000 and *Pst-avrRpm1* do not primarily stem from alterations in central metabolism. Rather, they likely involve more complex regulatory mechanisms specific to plant–pathogen interactions, warranting further investigation.

Overall, our comparative analysis of acetylation and succinylation modifications between AIPD and AIPA reveals marked differences. These disparities are evident in the inconsistent modification sites and the varied abundance patterns observed in co-modifications when compared to the control group. Acetylation modifications demonstrate conserved sequence features, whereas succinylation exhibits significantly reduced sequence specificity. Furthermore, the enrichment of acidic amino acids flanking the modification sites is not pronounced. Drawing from the enrichment analysis that integrates both acetylation and succinylation data, we formulate an initial hypothesis that the differentially modified proteins predominantly participate in the photosynthesis pathway and are tightly associated with light-harvesting proteins. This leads us to speculate that one potential factor contributing to the differences in pathogenicity between AIPD and AIPA could be their distinct mechanisms or modes of influencing photosynthesis. This intriguing possibility warrants further investigation. To validate and refine our understanding of these modifications and their potential role in pathogenicity, a focused approach involving the detailed analysis of specific proteins is warranted. Such studies have the potential to elucidate the precise mechanisms that underlie the observed modifications and how they contribute to the pathogenicity phenotypes associated with AIPD and AIPA. In conclusion, our findings provide a foundation for future research aimed at deciphering the complex regulatory networks that govern these modifications and their implications for plant health and disease.

## 5. Conclusions

In summary, Kac and Ksu modification sites in *Arabidopsis* infected with virulent *Pst* DC3000 and avirulent *Pst-avrRpm1* exhibit distinct preferences. Particularly, acetylation modifications displayed conserved sequence motifs, contrasting with the markedly reduced sequence specificity observed in succinylation events. The analysis of amino acids adjacent to these modification sites did not reveal a significant enrichment of acidic residues, suggesting a complex modulation of these post-translational modifications. By integrating acetylation and succinylation data, it is hypothesized that proteins carrying these modifications play pivotal roles in the photosynthesis pathway, particularly in light-harvesting complexes. Variations in pathogenicity might arise from divergent mechanisms by which acetylation and succinylation affect photosynthetic processes. This intriguing prospect warrants further exploration and may shed new light on the intricate regulatory networks governing plant–pathogen interactions.

## Data Availability

Data are contained within the article.

## Supplementary Materials

The following supporting information can be downloaded at: https://www.mdpi.com/article/10.3390/genes15040499/s1. Figure S1: Scatter plot of the mass error distribution of lysine modification sites. Figure S2: Two-dimensional distribution map of the length of the peptide. Figure S3: Number distribution of modification sites per peptide. Figure S4: GO-based enrichment analysis of identified down-regulated differentially modified Kac and Ksu proteins. Table S1: Motif analysis of the detected lysine acetylation. Table S2: Motif analysis of the detected lysine succinylation. Table S3: GO enrichment analyses of the identified lysine acetylated. Table S4: GO enrichment analyses of the identified lysine succinylated. Table S5: Enrichment analysis of KEGG pathways associated with down-regulated differentially modified Kac proteins. Table S6: Enrichment analysis of KEGG pathways associated with down-regulated differentially modified Ksu proteins. Table S7: Protein domain enrichment analysis of acetylated proteins. Table S8: Protein domain enrichment analysis of succinylated proteins. Table S9: GO-based enrichment analysis of identified down-regulated differentially modified Kac proteins. Table S10: GO-based enrichment analysis of identified down-regulated differentially modified Ksu proteins.

## References

[1] Fisher M.C., Henk D.A., Briggs C.J., Brownstein J.S., Madoff L.C., McCraw S.L., Gurr S.J. (2012). Emerging Fungal Threats to Animal, Plant and Ecosystem Health. Nature.

[2] Mansfield J., Genin S., Magori S., Citovsky V., Sriariyanum M., Ronald P., Dow M., Verdier V., Beer S.V., Machado M.A. (2012). Top 10 Plant Pathogenic Bacteria in Molecular Plant Pathology. Mol. Plant Pathol..

[3] Bent A.F., Mackey D. (2007). Elicitors, Effectors, and R Genes: The New Paradigm and A Lifetime Supply of Questions. Annu. Rev. Phytopathol..

[4] Lim C.W., Luan S., Lee S.C. (2014). A Prominent Role for RCAR3-mediated ABA Signaling in Response to *Pseudomonas syringae* pv. tomato DC3000 Infection in Arabidopsis. Plant Cell Physiol..

[5] de la Torre F., Gutiérrez-Beltrán E., Pareja-Jaime Y., Chakravarthy S., Martin G.B., del Pozo O. (2013). The Tomato Calcium Sensor Cbl10 and Its Interacting Protein Kinase Cipk6 Define a Signaling Pathway in Plant Immunity. Plant Cell.

[6] Alfano J.R., Collmer A. (2004). Type III Secretion System Effector Proteins: Double Agents in Bacterial Disease and Plant Defense. Annu. Rev. Phytopathol..

[7] Thomma B.P., Nurnberger T., Joosten M.H. (2011). Of PAMPs and Effectors: The Blurred PTI-ETI Dichotomy. Plant Cell.

[8] Eitas T.K., Dangl J.L. (2010). NB-LRR Proteins: Pairs, Pieces, Perception, Partners, and Pathways. Curr. Opin. Plant Biol..

[9] Yoon M., Middleditch M.J., Rikkerink E.H.A. (2022). A Conserved Glutamate Residue in RPM1-INTERACTING PROTEIN4 is ADP-ribosylated by the *Pseudomonas* Effector AvrRpm2 to activate RPM1-mediated plant resistance. Plant Cell.

[10] Carabetta V.J., Greco T.M., Cristea I.M., Dubnau D. (2019). YfmK is An Nε-lysine Acetyltransferase That Directly Acetylates the Histone-like Protein Hbsu in *Bacillus subtilis*. Proc. Natl. Acad. Sci. USA.

[11] Ren J., Sang Y., Lu J., Yao Y.F. (2017). Protein Acetylation and Its Role in Bacterial Virulence. Trends Microbiol..

[12] Li J., Ma X., Wang C., Liu S., Yu G., Gao M., Qian H., Liu M., Luisi B.F., Gabriel D.W. (2022). Acetylation of A Fungal Effector that Translocates Host PR1 Facilitates Virulence. eLife.

[13] Xie L., Li W., Xie J. (2012). Prokaryotic Nε-lysine Acetylomes and Implications for New Antibiotics. J. Cell. Biochem..

[14] Hu L.I., Lima B.P., Wolfe A.J. (2010). Bacterial Protein Acetylation: The Dawning of a New Age. Mol. Microbiol..

[15] Narita T., Weinert B.T., Choudhary C. (2019). Functions and Mechanisms of Non-histone Protein Acetylation. Nat. Rev. Mol. Cell Biol..

[16] Yang X.J., Seto E. (2008). Lysine Acetylation: Codified Crosstalk with Other Posttranslational Modifications. Mol. Cell.

[17] Weinert B.T., Schölz C., Wagner S.A., Iesmantavicius V., Su D., Daniel J.A., Choudhary C. (2013). Lysine Succinylation is A Frequently Occurring Modification in Prokaryotes and Eukaryotes and Extensively Overlaps with Acetylation. Cell Rep..

[18] Wagner S.A., Beli P., Weinert B.T., Nielsen M.L., Cox J., Mann M., Choudhary C. (2011). A Proteome-wide, Quantitative Survey of in Vivo Ubiquitylation Sites Reveals Widespread Regulatory Roles. Mol. Cell. Proteom..

[19] Sun L., Yao Z., Guo Z., Zhang L., Wang Y., Mao R., Lin Y., Fu Y., Lin X. (2019). Comprehensive Analysis of the Lysine Acetylome in *Aeromonas hydrophila* Reveals Cross-talk between Lysine Acetylation and Succinylation in LuxS. Emerg. Microbes Infect..

[20] Zhang Z., Tan M., Xie Z., Dai L., Chen Y., Zhao Y. (2011). Identification of lysine succinylation as a new post-translational modification. Nat. Chem. Biol..

[21] Zhen S., Deng X., Wang J., Zhu G., Cao H., Yuan L., Yan Y. (2016). First Comprehensive Proteome Analyses of Lysine Acetylation and Succinylation in Seedling Leaves of *Brachypodium distachyon* L.. Sci. Rep..

[22] Zhou X., Qian G., Yi X., Li X., Liu W. (2016). Systematic Analysis of the Lysine Acetylome in *Candida albicans*. J. Proteom. Res..

[23] Zheng H., He Y., Zhou X., Qian G., Lv G., Shen Y., Liu J., Li D., Li X., Liu W. (2016). Systematic Analysis of the Lysine Succinylome in *Candida albicans*. J. Proteom. Res..

[24] Zhang W., Jiang L.H., Huang J., Ding Y.Q., Liu Z.B. (2020). Loss of Proton/calcium Exchange 1 Results in the Activation of Plant Defense and Accelerated Senescence in *Arabidopsis*. Plant Sci..

[25] Huang D.W., Sherman B.T., Tan Q., Kir J., Liu D., Bryant D., Guo Y.J., Stephens R., Baseler M.W., Lane H.C. (2007). DAVID Bioinformatics Resources: Expanded Annotation Database and Novel Algorithms to Better Extract Biology from Large Gene Lists. Nucleic Acids Res..

[26] Xing E., Fan X., Jiang F., Zhang Y. (2023). Advancements in Research on Prevention and Control Strategies for Maize White Spot Disease. Genes.

[27] Li C., Zhang L., Ji H., Song W., Zhong Z., Jiang M., Zhang Y., Li Q., Cheng L., Kou M. (2023). RNA-Sequencing Analysis Revealed Genes Associated with Sweet Potato (*Ipomoea batatas* (L.) Lam.) Responses to Stem Rot during Different Infection Stages. Genes.

[28] Ronald P.C., Beutler B. (2010). Plant and Animal Sensors of Conserved Microbial Signatures. Science.

[29] DeFalco T.A., Zipfel C. (2021). Molecular Mechanisms of Early Plant Pattern-triggered Immune Signaling. Mol. Cell.

[30] Xia L., Kong X., Song H., Han Q., Zhang S. (2021). Advances in Proteome-wide Analysis of Plant Lysine Acetylation. Plant Commun..

[31] Zhang Y., Wang G., Song L., Mu P., Wang S., Liang W., Lin Q. (2017). Global Analysis of Protein Lysine Succinylation Profiles in Common Wheat. BMC Genom..

[32] Melo-Braga M.N., Verano-Braga T., Leon I.R., Antonacci D., Nogueira F.C.S., Thelen J.J., Larsen M.R., Palmisano G. (2012). Modulation of Protein Phosphorylation, *N*-glycosylation and Lys-acetylation in Grape (*Vitis vinifera*) Mesocarp and Exocarp Owing to *Lobesia botrana* Infection. Mol. Cell. Proteom..

[33] Walley J.W., Shen Z., McReynolds M.R., Schmelz E.A., Briggs S.P. (2018). Fungal-Induced Protein Hyperacetylation in Maize Identified by Acetylome Profiling. Proc. Natl. Acad. Sci. USA.

[34] Cao Y., Fan G., Wang Z., Gu Z. (2019). Phytoplasma-induced Changes in the Acetylome and Succinylome of *Paulownia tomentosa* Provide Evidence for Involvement of Acetylated Proteins in Witches’ Broom disease. Mol. Cell. Proteom..

[35] Yuan B., Liu T., Cheng Y., Gao S., Li L., Cai L., Yang J., Chen J., Zhong K. (2021). Comprehensive Proteomic Analysis of Lysine Acetylation in *Nicotiana benthamiana* after Sensing CWMV Infection. Front. Microbiol..

[36] Choudhary C., Kumar C., Gnad F., Nielsen M.L., Rehman M., Walther T.C., Olsen J.V., Mann M. (2009). Lysine Acetylation Targets Protein Complexes and Co-regulates Major Cellular Functions. Science.

[37] Wang Q., Zhang Y., Yang C., Xiong H., Lin Y., Yao J., Li H., Xie L., Zhao W., Yao Y. (2010). Acetylation of Metabolic Enzymes Coordinates Carbon Source Utilization and Metabolic Flux. Science.

[38] Zhao S., Xu W., Jiang W., Yu W., Lin Y., Zhang T., Yao J., Zhou L., Zeng Y., Li H. (2010). Regulation of Cellular Metabolism by Protein Lysine Acetylation. Science.

[39] Lehtimaki N., Koskela M.M., Mulo P. (2015). Posttranslational modifications of chloroplast proteins: An emerging field. Plant Physiol..

[40] He D., Wang Q., Li M., Damaris R.N., Yi X., Cheng Z., Yang P. (2016). Global Proteome Analyses of Lysine Acetylation and Succinylation Reveal the Widespread Involvement of both Modification in Metabolism in the Embryo of Germinating Rice Seed. J. Proteom. Res..

[41] Xu Y.X., Chen W., Ma C.L., Shen S.Y., Zhou Y.Y., Zhou L.Q., Chen L. (2017). Proteome and Acetyl-proteome Profiling of *Camellia sinensis* cv. ‘Anjin Baicha’ during Periodic Albinism Reveals Alterations in Photosynthetic and Secondary Metabolite Biosynthetic Pathways. Front. Plant Sci..

[42] Wang C., Ding Y., Wang W., Zhao X., Liu Y., Timko M.P., Zhang Z., Zhang H. (2022). Insights into Gene Regulation of Jasmonate-Induced Whole-Plant Senescence of Tobacco under Non-Starvation Conditions. Plant Cell Physiol..

[43] Takahashi H., Kanayama Y., Zheng M.S., Kusano T., Hase S., Ikegami M., Shah J. (2004). Antagonistic Interactions between the SA and JA Signaling Pathways in Arabidopsis Modulate Expression of Defense Genes and Gene-for-gene Resistance to Cucumber Mosaic Virus. Plant Cell Physiol..

[44] Banday Z.Z., Nandi A.K. (2018). *Arabidopsis thaliana* GLUTATHIONE-S-TRANSFERASE THETA 2 Interacts with RSI1/FLD to Activate Systemic Acquired Resistance. Mol. Plant Pathol..

[45] Zhou H., Finkemeier I., Guan W., Tossounian M.A., Wei B., Young D., Huang J., Messens J., Yang X., Zhu J. (2018). Oxidative Stress-triggered Interactions between the Succinyl- and Acetyl-proteomes of Rice Leaves. Plant Cell Environ..

