# Supplementary material for: Proteomic Analysis of Lysine Acetylation and Succinylation to Investigate the Pathogenicity of Virulent Pseudomonas syringae pv. tomato DC3000 and Avirulent Line Pseudomonas syringae pv. tomato DC3000 avrRpm1 on Arabidopsis thaliana
Source: Genes (Basel). 2024 Apr 16;15(4):499. doi: 10.3390/genes15040499 (PMC11050401; doi:10.3390/genes15040499)
Supplement: Supplementary file 1 [file genes-15-00499-s001.zip › Supplementary FIG S1-4.pdf]

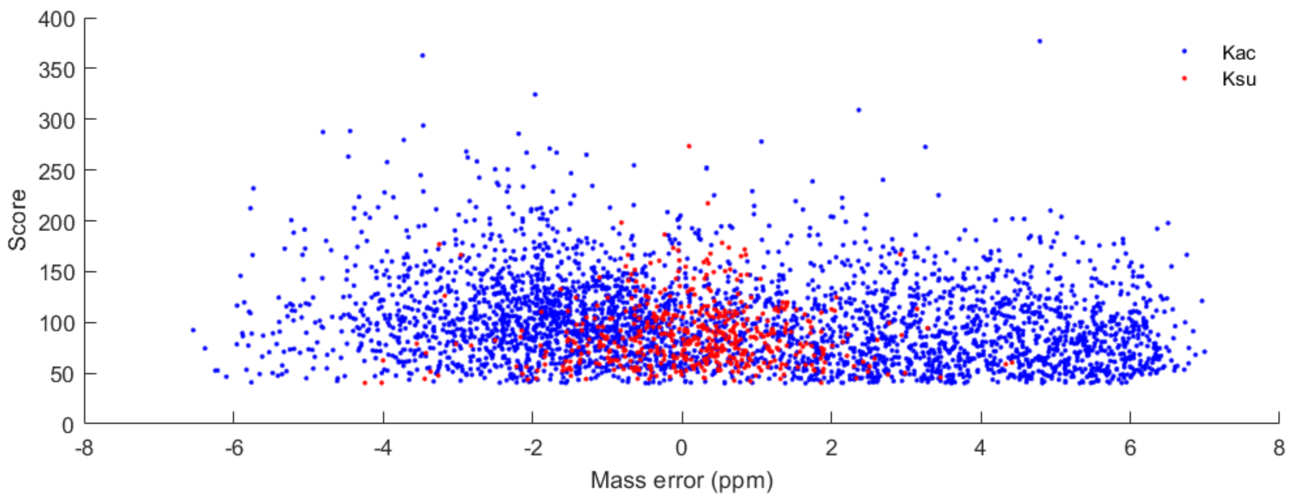

Figure S1. Scatter plot of the mass error distribution of lysine modification sites. Import all mass error data of sites into Matlab, and use Matlab to plot a scatter plot of the mass error distribution of lysine acetylation (Kac) sites and lysine succinylation (Ksu) sites.

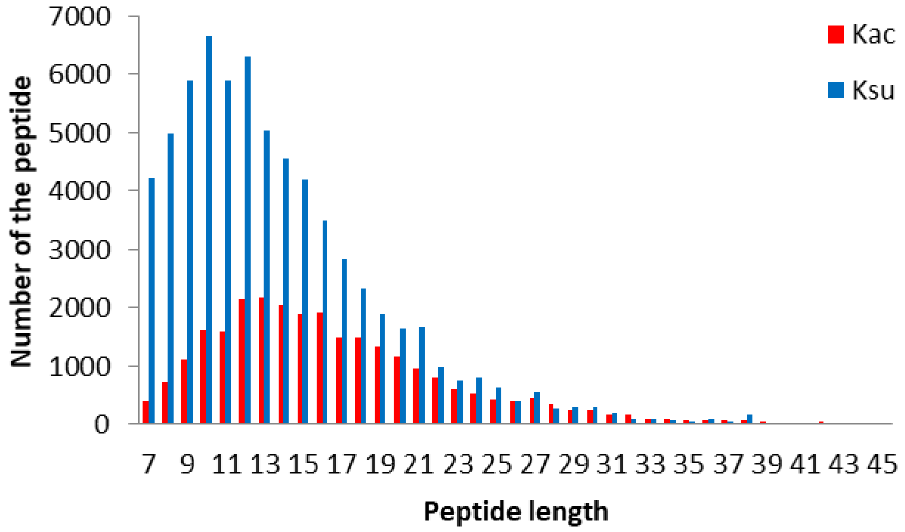

Figure S2. Two-dimensional distribution map of the length of the peptide.

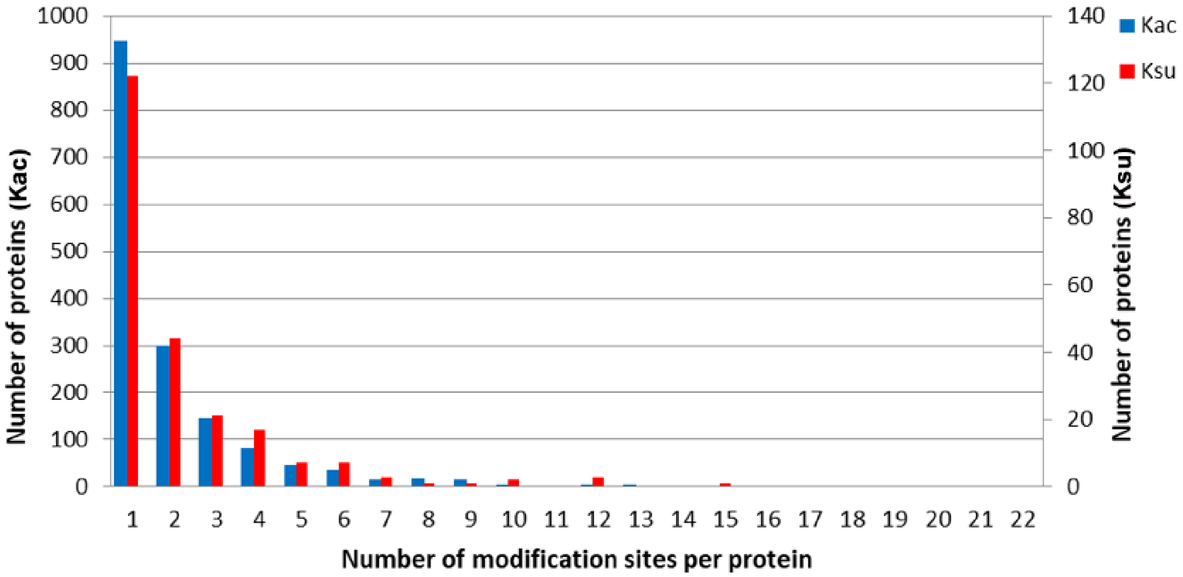

Figure S3. Number distribution of modification sites per peptide.

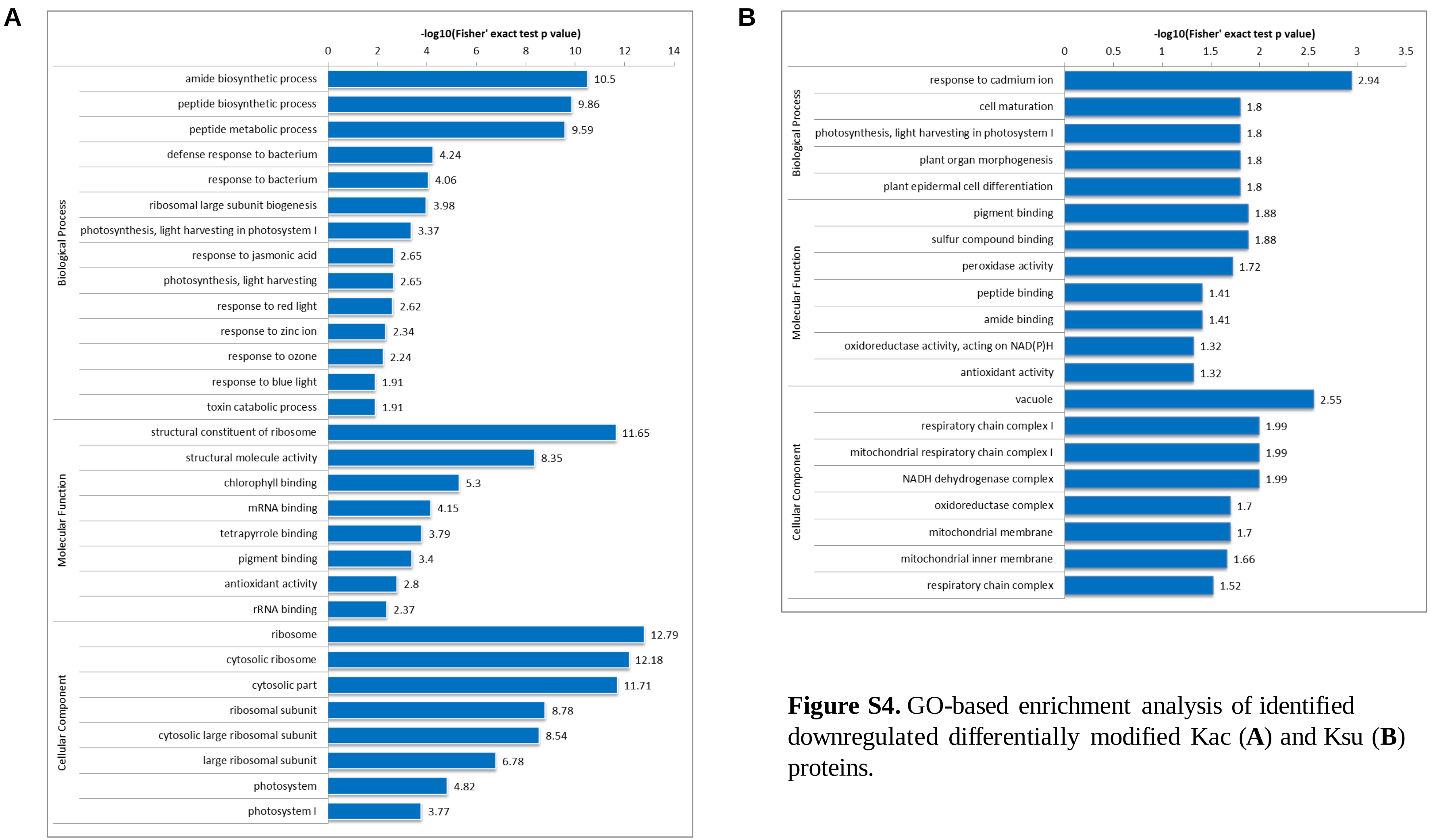

Figure S4. GO-based enrichment analysis of identified downregulated differentially modified Kac (**A**) and Ksu (**B**) proteins.
